# Sub-chronic nicotine exposure influences methamphetamine self-administration and dopamine overflow in a sex-and genotype-dependent manner in humanized *CHRNA*6 3′-UTR SNP (rs2304297) adolescent rats

**DOI:** 10.3389/fphar.2024.1445303

**Published:** 2024-08-14

**Authors:** Diana Carreño, Antonella Facundo, Anjelica Cardenas, Shahrdad Lotfipour

**Affiliations:** ^1^ Department of Pharmaceutical Sciences, University of California, Irvine, Irvine, CA, United States; ^2^ Department of Emergency Medicine, University of California, Irvine, Irvine, CA, United States; ^3^ Department of Radiation Oncology, David Geffen School of Medicine, University of California, Los Angeles, Los Angeles, CA, United States; ^4^ Department of Pathology and Laboratory Medicine, University of California, Irvine, Irvine, CA, United States

**Keywords:** electronic cigarettes, addiction, developing brain, microdialysis, tobacco, co-morbid drug use

## Abstract

**Introduction:** The rewarding effects of drugs of abuse are associated with the dopaminergic system in the limbic circuitry. Nicotine exposure during adolescence is linked to increased use of drugs of abuse with nicotine and methamphetamine (METH) commonly used together. Nicotine acts on neuronal nicotinic acetylcholine receptor (nAChR) systems, critical for reward processing and drug reinforcement, while METH leads to a higher dopamine (DA) efflux in brain reward regions. A human single nucleotide polymorphism (SNP) in the 3′-untranslated region (UTR) of the α6 nicotinic receptor subunit gene (*CHRNA*6, rs2304297), has been linked with tobacco/nicotine and general substance use during adolescence. Using CRISPR-Cas9 genomic engineering, our lab recapitulated the *CHRNA*6 3′UTR^C123G^ SNP, generating α6^CC^ and α6^GG^ allele carriers in Sprague Dawley rats. We hypothesized the *CHRNA*6 3′UTR^C123G^ SNP would sex- and genotype-dependently enhance nicotine-induced METH self-administration as well as nicotine-induced DA overflow in the nucleus accumbens shell of adolescent α6^GG^ and α6^CC^ carriers.

**Methods:** Adolescent male and female rats underwent a 4-day sub-chronic, low-dose (0.03 mg/kg/0.1 mL, x2) nicotine pretreatment paradigm to assess intravenous METH (0.02 mg/kg/0.1 mL) self-administration as well as nicotine- and METH (0.02 mg/kg/0.1 mL)-induced DA overflow in the nucleus accumbens shell (NAcS) using *in vivo* microdialysis coupled with high-performance liquid-chromatography-electrochemical detection (HPLC-ECD).

**Results:** Nicotine pretreatment sex- and genotype-dependently enhanced subsequent METH self-administration in adolescent *CHRNA*6 3′UTR^C123G^ SNP rats. Further nicotine and METH-induced DA overflow is observed in α6^CC^ females as compared to α6^GG^ females, with METH-induced DA overflow enhanced in α6^GG^ males when compared to α6^CC^ males.

**Conclusion:** These findings demonstrate that the *CHRNA*6 3′-UTR^C123G^ SNP can sex- and genotype-dependently impact adolescent nicotine-induced effects on METH self-administration and stimulant-induced DA overflow in reward regions of the brain.

## 1 Introduction

Adolescence is a critical period of development characterized by heightened susceptibility to environmental influences, including substance use. Nicotine and methamphetamine (METH) are two commonly abused substances that can have profound, long lasting consequences on the developing brain. Adolescents predominantly favor electronic cigarettes as their primary choice among tobacco products (Gorukanti et al., 2017). In 2023, the Annual National Youth Tobacco Survey reported more than one in four of current youth e-cigarette users use an e-cigarette product every day (Birdsey et al., 2023). Most e-cigarettes contain nicotine, a highly addictive substance that can harm the developing brain of adolescents (Goriounova and Mansvelder, 2012; Yuan et al., 2015). Adolescent exposure to nicotine may lead to the use of other tobacco products and drugs of abuse and amplify common underlying risk factors, i.e., genetic predisposition, peer influence, socioeconomic status and family history (McCabe et al., 2018; Berry et al., 2019; Ren and Lotfipour, 2019).

The neurobiological actions of nicotine are similar to other psychomotor stimulants including cocaine and METH (Laviolette and van der Kooy, 2004). Nicotine binds to nAChRs, leading to increased dopamine (DA) release and neurotransmitter modulation, resulting in enhanced alertness, reward and potential dependence (Di Chiara and Imperato, 1988; Albuquerque et al., 2009; Dani, 2015). METH is a potent analog of the stimulant, amphetamine. METH increases DA levels by inhibiting the reuptake and promoting release of DA, causing intense euphoria, heightened alertness, and potential neurotoxicity with chronic use (Rothman et al., 2001; Elkashef et al., 2008; Rusyniak, 2011; Ferrucci et al., 2019). It has been hypothesized that nicotine addiction is mediated by DA release in the mesolimbic system via local action at somatodendritic sites in the ventral tegmental area (VTA) (Di Chiara and Imperato, 1988; Nisell et al., 1994). Indeed, injections of 6-hydroxydopamine, a neurotoxin used to selectively destroy dopaminergic and noradrenergic neurons, into the nucleus accumbens (NAc) blocked nicotine self-administration in adult rats (Corrigall et al., 1994). *In vitro* studies have shown that METH evokes greater efflux of DA via the dopamine transporter (DAT) than its counterpart, amphetamine (Goodwin et al., 2009). *In vivo* studies have shown that nicotine pretreatment enhances the acquisition of METH self-administration and increases its intake in adolescent male, but not female rats (Dao et al., 2011; Cardenas and Lotfipour, 2022). Additionally, repeated administration of METH and nicotine in mice produced locomotor sensitization effects and a symmetrical cross-sensitization (Kuriban, 1999).

During adolescence, the α6 nAChR subunit reaches peak mRNA expression in dopaminergic cell bodies in the VTA and substantia nigra (SNg) (Azam et al., 2007). Moreover, the α6 nAChR subunit is localized in the mesolimbic and nigrostriatal DA pathway (Le Novère et al., 2002; Azam et al., 2007; Jackson et al., 2009) which may indicate a role of α6*-containing nAChRs in nicotine-induced behaviors and DA release (*denotes other associated nAChR subunits) (Le Novère et al., 2002; Azam et al., 2007; Pons et al., 2008; Jackson et al., 2009; Zoli et al., 2015). *In vivo* studies have demonstrated the α6* nAChRs have been shown to be involved in nicotine-induced locomotion (Drenan et al., 2008; Cardenas et al., 2022), nicotine self-administration and drug-seeking behavior (Pons et al., 2008; Brunzell et al., 2010; Carreño and Lotfipour, 2023; Carreño et al., 2024). Nevertheless, there is a scarcity of studies that examine the impact of α6* nAChRs in adolescent nicotine-induced behaviors. Despite this gap in research, it is important to note a robust correlation between tobacco/nicotine intake during adolescence and subsequent METH use (Brecht et al., 2007; Russell et al., 2008). Repeated self-administration of METH upregulated the *CHRNA*6 mRNA in the VTA of adult male rats (Bosch et al., 2015). Given these results, exploring how nicotine affects METH self-administration and the underlying mechanisms for how nicotine and METH exposure impact DA overflow in the NAc shell in adolescent rodent lines carrying a human *CHRNA*6 3′-UTR SNP becomes crucial.

To model adolescent initiation of nicotine smoking behavior in rats, a low-dose 4-day intravenous nicotine pretreatment paradigm was used. Previously, this paradigm has shown nicotine-induced acquisition of subsequent cocaine, fentanyl, METH, and alcohol in adolescent rats. (McQuown et al., 2007; Dao et al., 2011; Linker et al., 2020; Cardenas et al., 2021). Following nicotine treatment, animals underwent 2-h operant intravenous METH self-administration over 5-days (Dao et al., 2011; Cardenas and Lotfipour, 2022). Given our prior results that nicotine exposure can sex- and age-dependently enhance METH self-administration (Cardenas and Lotfipour, 2022), our current studies evaluated the combined interactions in our *CHRNA*6 3′UTR SNP rat lines in order to test the hypothesis that α6*-containing nAChRs mechanisms influence these relationships. In parallel, we used the same 4-day sub-chronic paradigm to evaluate nicotine- and METH-induced DA overflow in the NAc shell in adolescent male and female *CHNRA*6 3′-UTR SNP rats. Our recent studies illustrate that adolescent nicotine reinstatement behavior predicts DA tissue levels in *CHRNA*6 3′UTR SNP rat lines providing support for our hypothesis (Carreño et al., 2024). To better understand mechanisms underlying our observed effects, male and female adolescent rats (PN 31) underwent *in vivo* analytical quantification of neurotransmitters collected from the interstitial fluid from the NAc shell. DA, and its metabolites, 3,4-Dihydroxyphenylacetic acid (DOPAC), and homovanillic acid (HVA) from the extracellular space were measured by high performance liquid chromatography coupled with electrochemical detection (HPLC-ECD) methods. Our studies test the hypothesis that sex- and genotype-dependent effects will be observed for nicotine-induced METH self-administration and drug-induced DA overflow in adolescent *CHRNA*6 3′-UTR SNP rats. The objective of this study is to fill critical gaps in understanding how nicotine exposure during adolescence and the *CHRNA*6 3′-UTR SNP influences METH self-administration and associated neurobiological changes. By exploring sex- and genotype-dependent effects, we gain insights into the complex interplay between genetics, sex, and co-substance use in adolescents, thereby informing future strategies for the prevention and intervention of substance abuse disorders.

## 2 Methods

### 2.1 Animals

Male and female, *CHRNA*63′-UTR SNP knock-in rats were developed and bred in house as previously described (Cardenas et al., 2022; Carreño and Lotfipour, 2023). Juveniles were weaned at postnatal day (PN 21), separated by sex, and handled for 3 days prior to experimentation. Rats were group-housed in a controlled 12-h light–dark cycle in an AAALAC-accredited vivarium. Food and water were available *ad libitum* except when indicated. Animals were weighed daily to ensure the maintenance of normal growth. To avoid potential litter effects, one pup per litter per experimental group was used for data collection. Animals were allocated to experimental groups using a random sequence generator. The study was conducted according to the guidelines of the Institutional Animal Care and Use Committee (IACUC) at the University of California, Irvine (UCI) (protocol: AUP-21-022, 4 February 2021). UCI’s IACUC has Animal Welfare Assurance #D16-00259 (#A3416.01) approval on file with the Office of Laboratory Animal Welfare at NIH and is fully accredited by AAALAC, International. *CHRNA*6 3′UTR SNP rat strains (SD-*CHRNA*6^em1Slot^ 984, SD-*CHRNA*6^em2Slot^ 985) have been donated to the Rat Resource and Research Center (RRRC).

### 2.2 Drugs

Nicotine tartrate (Glentham Life Sciences, Corsham, Wiltshire, United Kingdom) was calculated as a base, dissolved in saline, with a final pH of 7.2–7.4. Methamphetamine (METH) (National Institute of Drug Abuse (NIDA), Bethesda, MD) was dissolved in saline and filtered via 0.22 μm sterile filters (VWR). Equithesin (Pentobarbital and Chloral Hydrate (Sigma Aldrich, St. Louis, MO, United States), propylene glycol, ethanol and magnesium sulfate) is made in the laboratory, similar to our prior studies (Carreño and Lotfipour, 2023; Carreño et al., 2024). Carprofen (Zoetis, Parsippany, NJ, United States) was diluted in saline. Nicotine, Equithesin, and carprofen were filtered via 0.22 μm sterile filters (VWR, Radnor, PA, United States). Propofol (Medvet, Mettawa, IL, United States) in a 5 mg/kg, intravenous (i.v.) was administered to test for catheter patency.

### 2.3 Surgical implantation of intravenous catheter

**FIGURE 1:**
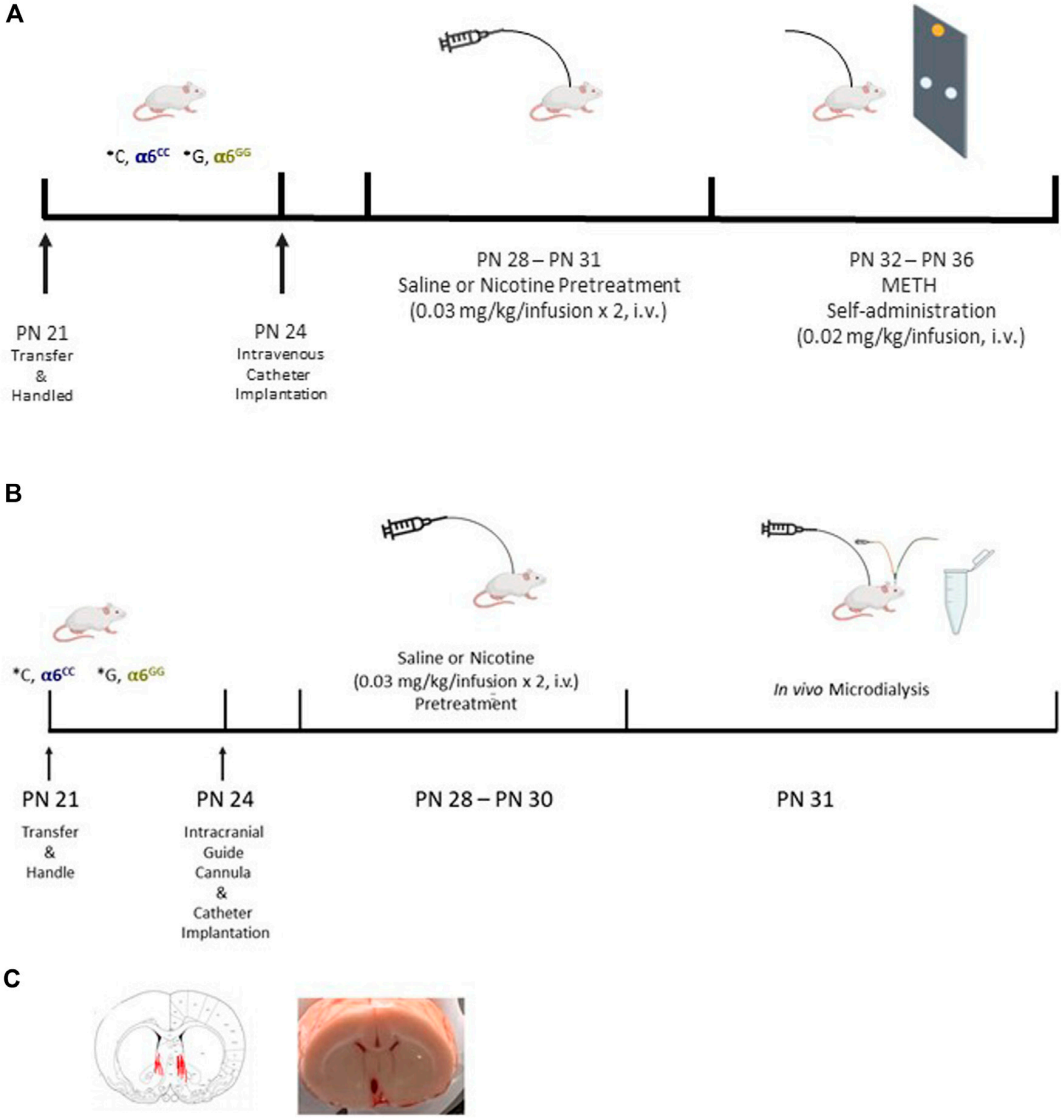
Experimental Design in the *CHRNA*6 3′UTR SNP Knock-In. Male and female *CHRNA*6 3′-UTR SNP knock-in rats were bred in-house and underwent a series of procedures including nicotine pretreatment and METH self-administration **(A)**. *In vivo* microdialysis was performed to assess dopamine and its metabolites after drug administration **(B)**. Probe placement confirmation in male and female *CHRNA*6 3′-UTR SNP knock-in rats **(C)**.

Catheter construction and implantation was done as described previously (Belluzzi et al., 2005). Animals were anesthetized with Equithesin (0.0035 mL/g body weight), and a chronic catheter was surgically implanted into the right external jugular vein. The catheter was passed subcutaneously from the animal’s back to the jugular vein where the tubing was inserted. The cannula assembly was affixed to the animal’s back and sealed to maintain an unobstructed system. Wound closures were made with wound clips, antiseptic ointment was applied to the incisions, and carprofen (5 mg/kg, subcutaneous) was injected to prevent infection. The animals were kept in a warm cage for post-surgical observation until they emerged from anesthesia. Catheter patency was tested for rapid (5–10 s) anesthesia by infusion of propofol (5 mg/kg) before and after completion of self-administration and *in vivo* microdialysis experiments (Figure 1).

### 2.4 Nicotine pretreatment

Starting on PN 28 rats were administered nicotine (2 × 0.03 mg/kg/0.1 mL, i.v.) or saline injections spaced 1 minute apart daily for 4 days (Figure 1A). The dual injection of 0.03 mg/kg nicotine daily over 4 days was chosen to model early initiation of nicotine use during adolescence (Cardenas et al., 2021), models prior studies using the same paradigm (Cardenas et al., 2021), and reduced receptor desensitization and toxic effects (McQuown et al., 2007). Furthermore, the 0.6 mg/kg dose is the optimal dose regularly chosen in nicotine self-administration studies, is equivalent to 1-2 cigarettes worth of nicotine/day and produces 30 ng/mL peak plasma levels in adolescent and adult rats (Cao et al., 2007; Matta et al., 2007).

### 2.5 METH self-administration

One day after nicotine pretreatment, animals were tested in 28 × 25 × 30 cm self-administration chambers with two nose pokes holes for 5 days (PN 32–36) (Figure 1A). Adolescent animals underwent a 2-h nose poke session on a fixed ratio 1 (FR1) schedule to administer METH (0.02 mg/kg/infusion (inf)) (Dao et al., 2011; Cardenas and Lotfipour, 2021) with a time-out period of 20 s signaled by a cue light over the reinforced hole. During this time-out period, animals were restricted from receiving another reinforced response (inf) and the house light was turned off. Nose poke of the nonreinforced hole was scored but did not result in a signal or inf. To determine catheter patency, after the last intravenous self-administration session, rats were administered propofol, a rapid anesthetic (0.05 mL for adolescents, 0.1 mL for adults, i.v.). Propofol negative animals were removed from the study analyses.

### 2.6 *Surgery, in vivo* microdialysis, and HPLC-ECD

#### 2.6.1 Stereotaxic surgery

Following the placement of the i.v. Catheter, animals designated for microdialysis underwent the implantation of a cranial guide cannula (PN 24). Animals were placed in a stereotaxic frame (Stoelting Co., Chicago, Il, United States), their skulls revealed and drilled to expose the dura. A chronic guide cannula (Bioanalytical Systems, Inc., West Lafayette, IN) was stereotaxically implanted 2.0 mm above the target area, fixed to the skull with acrylic dental cement, and sealed with a dummy cannula. Anatomical coordinates for adolescent animals (PN 28-29) were determined from the adult atlas (Paxinos and Watson, 1989), then empirically identified in a preliminary experiment with histological confirmation. The following guide cannula coordinates were measured from the dura; nucleus accumbens shell AP, + 2.1 mm; ML, ± 0.6 mm; DV,-6 mm.

#### 2.6.2 *In Vivo* microdialysis procedure

Animals were given 3 days to recover after surgeries with daily handling after surgeries and catheters flushed daily. Beginning on PN 28, the animals received either saline (acute condition) or nicotine (sub-chronic condition) pretreatment (PN 28-31, Figure 1A). Intravenous catheter patency was tested by propofol 1 day before the experiment. On the experimental day (PN 31), the dummy cannula was replaced with a 2 mm microdialysis probe with a 30 kDa cut-off membrane (MD-2200; Bioanalytical Systems, Inc., West Lafayette, IN). The probe membrane extended 2 mm beyond the tip of the guide cannula. The quality of probes was tested *in vitro* before the experiment with an average recovery of 10% (data not shown). Microdialysis was carried out under a free-moving condition in Culex NxT with Raturn–Multi Animal (Bioanalytical Systems, Inc., West Lafayette, IN), with the probe continuously perfused with artificial cerebrospinal fluid (Ringers Solution 147 mM NaCl, 2.2 mM CaCl_2_, 4 mM KCl) at a constant flow rate of 1.1 mL/min delivered by a Empis infusion pump (CX-300; Bioanalytical Systems, Inc., West Lafayette, IN) (Figure 1B). Dialysate was collected into a Honeycomb refrigerated fraction collector (MD-1201; Bioanalytical Systems, Inc., West Lafayette, IN) that maintained the samples at 4°C. After 4-h perfusion to establish an equilibration between the probes’ internal and external environment, baseline samples were collected every 20 min for 60 min. When DA levels reached a stable baseline, animals were given two 0.100 mL injections (i.v.) of saline, 1 min apart. After 100 min, nicotine (0.03 mg/kg/0.100 mL injection, i.v.) was injected twice at a 1 min interval and samples were collected for another 200 min. After 220 min, METH (0.02 mg/kg/0.100 mL injection, i.v.) was injected twice at 1 min interval and samples were collected for another 100 min (Figure 1). DA and its metabolite levels were quantified by HPLC-ECD. The position of microdialysis probes was verified histologically and mapped onto relevant atlas sections (Paxinos and Watson, 1986) (Figure 1C).

#### 2.6.3 HPLC-ECD

Microdialysate samples (20 μL) were automatically injected by an ESA 542 refrigerated auto-sampler onto a 150 × 3 mm ODS C18 column (ESA Inc., Chelmsford MA) connected to an ESA 580 HPLC pump. The column was kept at 35°C and perfused by MD-TM mobile phase (ESA, Chelmsford, MA) at a rate of 0.6 mL/min. DA and metabolite levels were determined by an electrochemical ESA 5600 detector with an ESA 5011 microdialysis cell with the dominant potential of 160 mV. The sensitivity of the detector is 500 fg. Measurements were analyzed using CoulArray for Windows Software 2.0 (ESA Inc., Chelmsford, MA, United States). Standard curves were generated with catecholamine (ThermoScientific, Wlatham, MA), DOPAC, and HVA (Sigma-Aldrich, St Louis, MO) standards, and levels in experimental samples were determined from the curve and expressed as ng/20 μL injection (Equation 1), as there were no significant differences in probe recovery. Basal levels of DA and its metabolites were determined by averaging the samples before nicotine injection. Nicotine- and METH-induced changes in DA and DA metabolite levels were expressed as area under the curve (AUC) given by Equation 1 below.

$$\begin{array}{ll} AUC= & \left(\text{Drug }\text{Time }\text{Point }1-\text{Average }\text{Baseline}\right)\\ & +\left(\text{Drug }\text{Time }\text{Point }2-\text{Average }\text{Baseline}\right)\\ & +\left(\text{Drug }\text{Time }\text{Point }3-\text{Average }\text{Baseline}\right) \end{array}$$ (1)

### 2.7 Statistics

Data were analyzed with JMP statistical analysis software (SAS Institute, Cary, NC). A total of 186 rats were used in our studies. Animals that did not exhibit anesthesia from propofol were removed from the analysis (n = 41). Further exclusions were applied to animals that fell outside the boundaries defined by predetermined box and whisker plots for each group (n = 6 for METH self-administration and n = 8 for microdialysis). The 5-day METH acquisition mean response data over days were analyzed by a repeated measure five-way analysis of variance (ANOVA) for sex × genotype × pretreatment × response × day, with a repeated measure on day and response. Microdialysate samples AUC were analyzed by a four-way ANOVA for sex x genotype x pretreatment (acute vs. sub-chronic) x drug (nicotine, METH, saline) with repeated measures for drug. Significant main effects or interactions were further tested by *t*-test with the Bonferroni adjustment for multiple comparisons.

## 3 Results

### 3.1 Nicotine pretreatment enhances subsequent METH self-administration in the *CHRNA*6 3′-UTR SNP knock-in rats in a sex- and genotype-dependent manner

**FIGURE 2:**
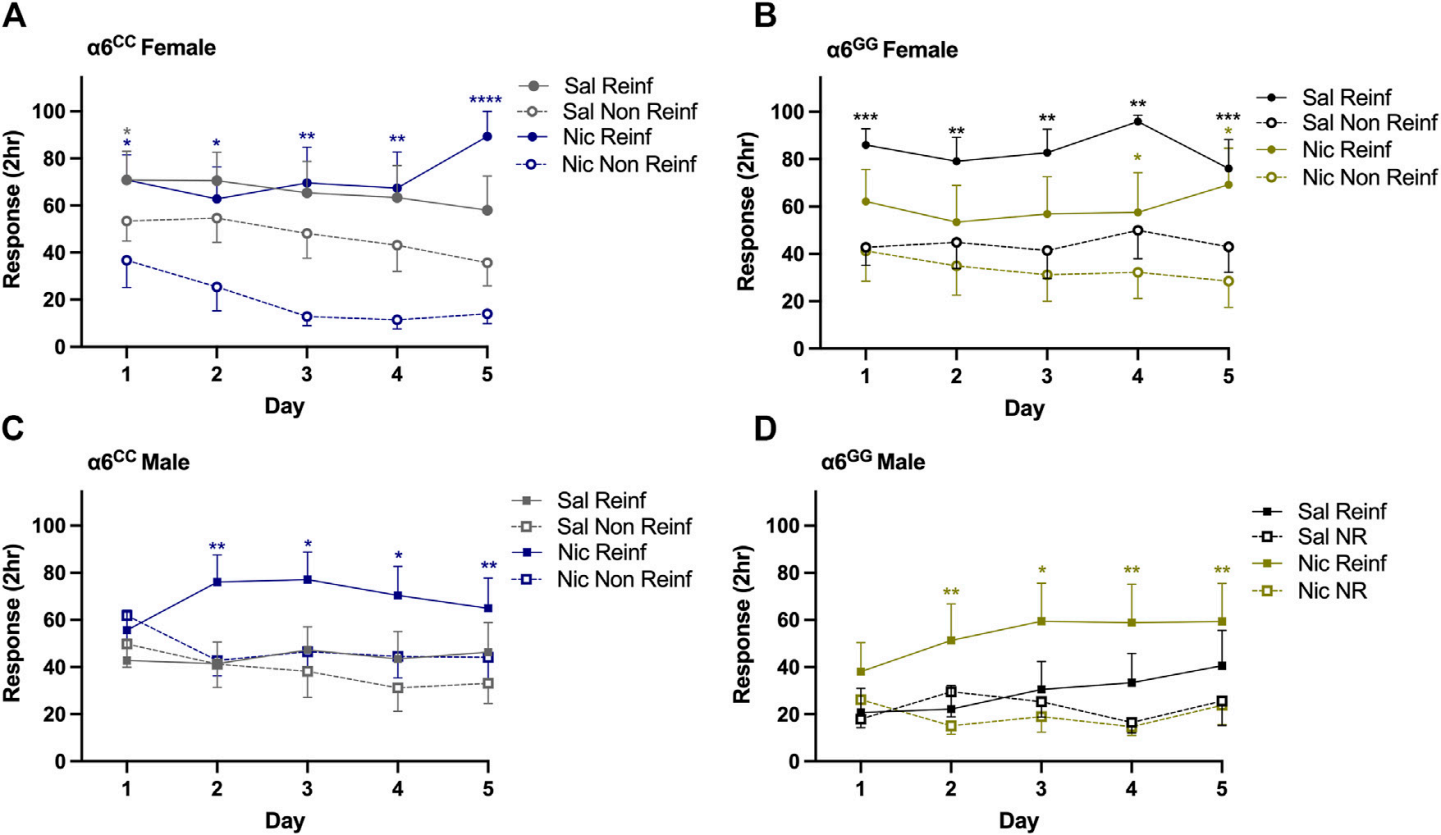
Sex- and genotype-dependent nicotine-induced METH self-administration in male and female *CHRNA*6 3′-UTR SNP rats. METH self-administration mean 2-h responses ±SEM across 5 days in **(A)** α6^CC^ **(B)** α6^GG^ females, **(C)** α6^CC^ and **(D)** α6^GG^ males. Nicotine-treated α6^CC^ and saline-treated α6^GG^ females maintain consistent METH self-administration, saline-pretreated α6^CC^ females initially prefer reinforced responding. Nicotine-treated males show sustained METH self-administration from days 2–5, independent of genotype. Open circles represent female data; open and closed squares represent male data. ****p < 0.0001, ***p < 0.001, **p < 0.01, *p < 0.05 Reinf versus Non Reinf responses; n = 9/12/group. METH = methamphetamine, Reinf = Reinforced, Non Reinf = Non-Reinforced, Sal = Saline (grey/black), Nic = Nicotine (blue/gold).

To determine whether the human SNP in the 3′-UTR of the *CHRNA*6 gene influences nicotine-induced METH self-administration, we evaluated mean response data over time. Overall ANOVA showed a main effect for reinforced/nonreinforced response [F_1,72_ = 41.86, *p* = 0.0001] with sex × reinforced/nonreinforced [F_1,72_ = 6.58, *p* = 0.012], pretreatment × reinforced/nonreinforced [F_1,72_ = 7.05, *p* = 0.0098], genotype × sex × pretreatment × reinforced/nonreinforced [F_1,72_ = 4.35, *p* = 0.04], response × day [F_4,288_ = 7.48, *p* = 0.0001], response × day × sex × pretreatment [F_4,288_ = 2.44, *p* = 0.047] and day × genotype [F_4,288_ = 2.47, *p* = 0.045] interactive effects. Since we identified interactions for every measure, we separated the data by genotype, sex, pretreatment, response, and day (Figures 2A–D). For females, nicotine-treated α6^CC^ and saline-treated α6^GG^ animals acquired and maintained METH self-administration on days 1–5 as measured by preference for reinforced over non-reinforced nose pokes (*p* < 0.05, Figures 2A, B). Conversely, saline-treated α6^CC^ female rats demonstrated preference for reinforced over nonreinforced nose poke on day 1 of METH self-administration (*p* = 0.023) whereas nicotine-treated α6^GG^ female rats acquired METH self-administration on days 4–5 (*p* < 0.05, Figures 2A, B). Nicotine-treated males responded for METH significantly more than saline-treated males on days 2–4, independent of genotype (*p* < 0.05, Figures 2C, D). Taken together, our findings demonstrate that adolescent sub-chronic nicotine exposure influences the acquisition and maintenance of METH in a sex-and genotype-dependent manner.

### 3.2 Nicotine pretreatment impacts drug-induced DA overflow in a sex- and genotype-dependent manner in the *CHRNA*6 3′-UTR SNP rats

**FIGURE 3:**
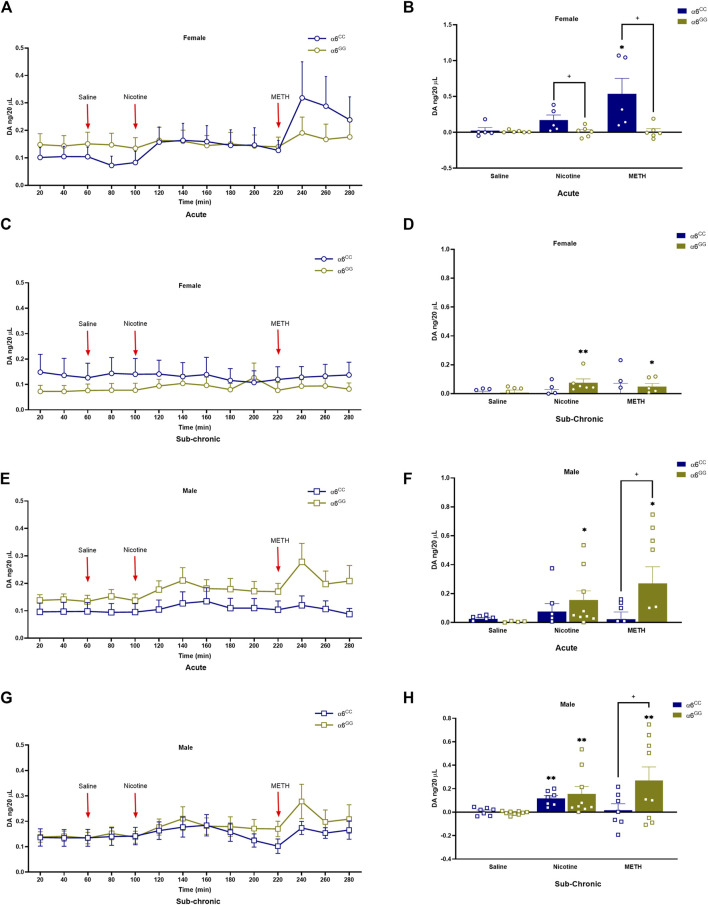
DA levels in male and female in *CHRNA*6 3′-UTR SNP Rats. The effects of nicotine- and METH-induced DA overflow in the NAc shell, were assessed in male and female adolescent (PN 31) *CHRNA*6 3′-UTR SNP rats. Female **(A)** acute timeline and **(B)** AUC **(C)** sub-chronic timeline and **(D)** AUC for saline, nicotine, and METH; Male **(E)** acute timeline and **(F)** AUC **(G)** sub-chronic timeline and **(H)** AUC for saline, nicotine, and METH. Nicotine and METH induce greater DA overflow in α6^CC^ females than α6^GG^ females in the acute condition, while α6^GG^ males show enhanced METH-induced DA overflow only acute condition. No significant genotype differences are noted in nicotine-induced DA overflow in males under either acute or sub-chronic conditions.**p < 0.01 vs. saline; *p < 0.05 vs. saline; +*p* < 0.05 α6^CC^ vs. α6^GG^. AUC = area under the curve METH = Methamphetamine N = 5-12/group.

The effects of nicotine- and METH-induced DA overflow in the NAc shell, were assessed in male and female adolescent (PN 31) *CHRNA*6 3′-UTR SNP rats. An overall ANOVA for DA overflow showed a between main effect for pretreatment [F_1,46_ = 5.9267, *p* = 0.0189], as well as between interactive effects for genotype x sex [F_1,46_ = 7.3049, *p* = 0.0096], genotype x sex x pretreatment [F_1,46_ = 8.6829, *p* = 0.0050]. In addition, we observed a within main effect for drug [F_2,92_ = 12.1493], as well as within interactive effects for drug x genotype x sex [F_2,92_ = 7.3632, *p* = 0.0011], drug x pretreatment [F_2,92_ = 4.9620, *p* = 0.0090], and drug x genotype x sex x pretreatment [F_1,92_ = 6.2993, *p* = 0.0027]. Thus, we evaluated these parameters separately. Nicotine and METH induced DA overflow is enhanced in α6^CC^ females as compared to α6^GG^ females (*p* < 0.05), primarily in acute condition (Figures 3A–D). METH induced DA overflow is enhanced in α6^GG^ males in the acute condition, but not sub-chronic condition. No variation in genotype were noted for nicotine effects in both acute and sub-chronic conditions in males. (Figures 3E–H).

**FIGURE 4:**
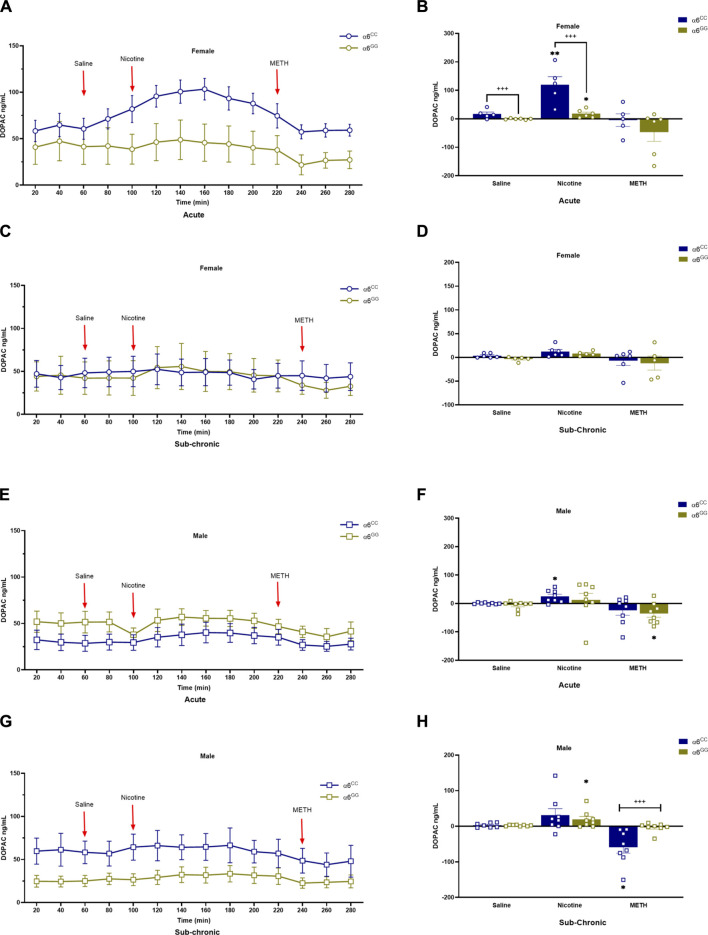
DOPAC levels in male and female in *CHRNA*6 3′-UTR SNP Rats. Female **(A)** acute timeline and **(B)** AUC **(C)** sub-chronic timeline and **(D)** AUC for saline, nicotine, and METH; Male **(E)** acute timeline and **(F)** AUC **(G)** sub-chronic timeline and **(H)** AUC for saline, nicotine, and METH. α6^CC^ females exhibit higher DOPAC levels than α6^GG^ females in response to saline and nicotine pretreatments, indicating genotype-specific DA metabolism in females. In males, this effect emerges under sub-chronic METH treatment, with α6^GG^ males showing higher DOPAC levels than α6^CC^ males. During acute conditions, α6^CC^ females display significantly higher DOPAC overflow for both saline and nicotine compared to α6^GG^ females (*p* < 0.001), a disparity not seen in males. Sub-chronic nicotine-treated α6^GG^ males exhibit greater METH-induced DOPAC overflow than α6^CC^ males. **p < 0.01, *p < 0.05 vs. saline; +++*p* < 0.001 α6^CC^ vs. α6^GG^. AUC = area under the curve; METH = Methamphetamine N = 5-12/group.

**FIGURE 5:**
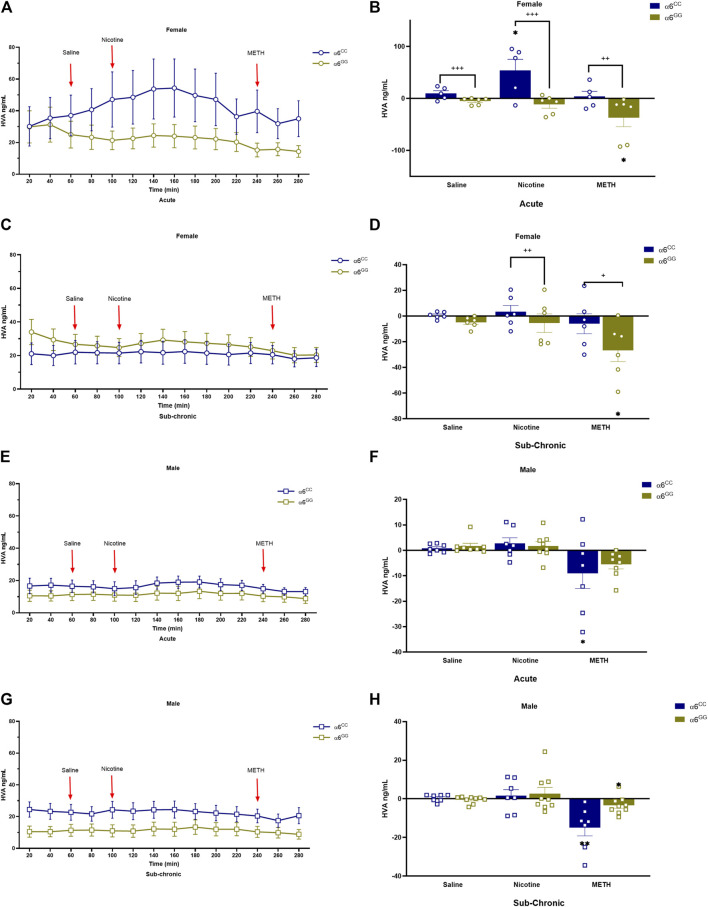
Figure 6 HVA levels in male and female in *CHRNA*6 3′-UTR SNP Rats. Female **(A)** acute timeline and **(B)** AUC **(C)** sub-chronic timeline and **(D)** AUC for Sal, Nic, and METH; Male **(E)** acute timeline and **(F)** AUC **(G)** sub-chronic timeline and **(H)** AUC for saline, nicotine, and METH. Saline- and nicotine-treated α6^CC^ females show altered HVA overflow compared to α6^GG^ females, a difference not observed in males **p < 0.01, *p < 0.05 vs. saline; +++*p* < 0.001 α6^CC^ vs. α6^GG^. AUC = area under the curve, METH = Methamphetamine N = 5-12/group.

We assessed DA metabolites, DOPAC and HVA, to determine their change in release in acute and sub-chronic condition. For DOPAC, a between main effect for pretreatment [F_1,52_ = 4.6560, *p* = 0.0356], in addition a between interactive effects for genotype x sex [F_1,52_ = 13.2859, *p* = 0.0006] and genotype x pretreatment [F_1,52_ = 12.0334, *p* = 0.0011] were observed. A 4-way multivariate ANOVA revealed drug x genotype x sex x genotype [F_2,104_ = 4.4833, *p* = 0.0136] interactive effects (Figures 4A–H). Data were separated by sex, genotype and pretreatment. In the acute condition, α6^CC^ females displayed higher DOPAC overflow for saline and nicotine for the acute condition as compared to α6^GG^ females (*p* < 0.001), this distinction was not evident in males (Figures 4A–F). In the sub-chronic condition, α6^GG^ males exhibited a greater METH-induced DOPAC overflow compared to α6^CC^ males (*p* < 0.001) (Figures 4G, H). HVA, an extracellular DA metabolite showed a between main effect for genotype [F_1,47_ = 12.4396, *p* = 0.0010], and between interactive effects for genotype x sex [F_1,47_ = 19.5262, *p* = 0.001], genotype x pretreatment [F_1,47_ = 7.2047, *p* = 0.01] and genotype x sex x pretreatment [F_1,47_ = 4.0431, *p* = 0.0501]. A 4-way multivariate ANOVA revealed drug x sex x genotype × pretreatment interaction [F_2,98_ = 3.7283, *p* = 0.0313]. Data were separated for sex, genotype and pretreatment. Nicotine and METH altered HVA overflow in saline- and nicotine-treated α6^CC^ females as compared to α6^GG^ females, these effects were not observed in males (Figures 5A–H).

## 4 Discussion

In the present investigation, the impact of the *CHRNA*6 3′-UTR SNP on nicotine-induced METH self-administration and the overflow of DA in the NAc shell revealed multiple interactions, indicating an intricate interrelationship involving sex, pretreatment, and genotype. Notably, nicotine-treated α6^CC^ and saline-treated α6^GG^ females exhibited METH self-administration over several days. α6^CC^ females when pretreated with saline (and not nicotine) initially showed a preference for reinforced versus non-reinforced responding for Day 1 METH self-administration. Nicotine-treated males, regardless of genotype, exhibited METH self-administration on days 2–5. These results highlight the sex- and genotype-dependent effects of adolescent sub-chronic nicotine exposure on the acquisition and maintenance of METH self-administration in rats with the human *CHRNA*6 3′-UTR SNP. Moreover, nicotine- and METH-induced DA overflow are enhanced in α6^CC^ females as compared to α6^GG^ females primarily in acute condition whereas α6^GG^ males showed enhanced METH-induced DA overflow in acute, but not sub-chronic condition. No genotype differences were observed for males in either acute- or sub-chronic condition for nicotine-induced DA overflow. Taken together, these findings illustrate the complex interplay of sex, genotype, and pretreatment in influencing the effects of nicotine and METH on DA dynamics and self-administration behavior.

### 4.1 The influence of nicotine pre-treatment on METH self-administration in the *CHRNA*6 3′-UTR knock-in rats

Previous behavioral studies in rodents with the *CHRNA*6 3′-UTR SNP knock-in have shown that α6^CC^ females and α6^GG^ males displayed nicotine-induced enhanced locomotor and anxiolytic behavior when compared to their saline-treated counterparts (Cardenas et al., 2022). In addition, nicotine-induced locomotion and anxiolytic behavior in α6^CC^ females was significantly higher than α6^GG^ females. In contrast, nicotine-induced anxiolytic behavior in males was significantly higher in α6^GG^ as compared to α6^CC^ males (Cardenas et al., 2022). In the current study, we illustrate additional sex- and genotype-dependent adolescent nicotine exposure effects on subsequent METH self-administration. Nicotine pretreatment enhanced self-administration for METH in α6^CC^ female rats. In contrast, saline-, but not nicotine-treated, α6^GG^ females show self-administration for METH. In comparison, Sprague Dawley WT adolescent females did not exhibit increased METH responsiveness, whether or not they received nicotine pretreatment (Cardenas and Lotfipour, 2021). In male adolescents, prior nicotine exposure increased subsequent METH self-administration, its maintenance, and intake, independent of *CHRNA*6 3′-UTR SNP genotype. *CHRNA*6 3′-UTR SNP knock-in adolescent male rat data replicates adolescent WT male nicotine-induced enhancement of METH self-administration (Dao et al., 2011; Cardenas and Lotfipour, 2022). Sex and nicotine effects have been observed for METH and amphetamine self-administration as well as stimulant locomotor sensitization which may be further impacted by genetics (Collins et al., 2004; Collins and Izenwasser, 2004; Harmony et al., 2020). A potential mechanism for developmental nicotine exposure effects on subsequent METH self-administration is regulation of α6* nAChRs. A bidirectional effect of sex on transcriptional regulation of α6 nAChR mRNA expression and nicotine behaviors has been observed though protein kinase C epsilon in adult mice (Moen et al., 2021). However, we do not observe baseline or nicotine-induced α6 nAChR subunit mRNA differences in the *CHRNA*6 3′-UTR SNP knock-in rats (Cardenas et al., 2022). Whether the *CHRNA*6 3′-UTR SNP impact the translation of the α6 nAChR subunit protein needs to be evaluated in future studies. However, we are limited by lack of α6 specific antibodies available (Cardenas et al., 2020). Either way, our current findings suggest functional changes of α6 nAChRs in reward regions of the brain based on the *CHRNA*6 3′-UTR SNP genotype, sex, and drug treatment effects observed in our studies. These findings emphasize the critical role of using translational models to understand the complex interplay of sex, genotype, and pretreatment in influencing nicotine and METH effects. This approach can help in developing more targeted and effective interventions for substance use disorders. While our current studies have focused on drug reinforcement and neurotransmitter overflow, future studies could evaluate the effects of natural rewards as well in the *CHRNA*6 3′UTR SNP rodent line. Indeed, we have previously illustrated that our adolescent humanized *CHRNA*6 3′UTR SNP rats do not differ based on natural food rewards (Carreño and Lotfipour, 2023). Further, prior studies in wild type Sprague Dawley rats have illustrated that the 4-day nicotine pretreatment paradigm has minimal impacts on sucrose taking behavior, with no impacts on extinction and reinstatement during adolescents or adults, which is different than what is observed for stimulants (Mojica et al., 2014). For these reasons, we have not assessed sucrose reinforcement, extinction and reinstatement in our studies. Nevertheless, future studies could evaluate this to confirm whether similar results would be observed in the *CHRNA*6 3′UTR SNP rats. Furthermore, future studies should investigate sex-, genotype-, and nicotine-dependent α6* nicotinic receptor protein expression via binding assays in tissue of humanized *CHRNA*6 3-UTR SNP rats.

### 4.2 Nicotine pretreatments effects on drug-induced DA overflow are impacted in a sex- and genotype-dependent manner in *CHRNA*6 3′-UTR SNP rats

We have previously shown sex, age and genotype difference of DA tissue levels in adolescent, adult and nicotine-seeking *CHRNA*6 3′-UTR SNP knock-in rats (Carreño et al., 2024). Post-reinstatement, male α6^GG^ rats show suppressed DA levels in the NAc shell compared to baseline (Carreño et al., 2024). The present study emphasizes the distinctions of DA transmission in the *CHRNA*6 3′-UTR SNP knock-in adolescent female and male rats of nicotine and METH. In the acute condition, α6^CC^ females demonstrated heighten DA overflow by nicotine- and METH as compared to the α6^GG^ females. In contrast, α6^GG^ males exhibited greater METH-induced DA overflow when compared to α6^CC^ males in the acute, but not sub-chronic condition, which may suggest tolerance effects. No genotype differences were observed for nicotine-induced DA overflow in the acute or sub-chronic condition in adolescent males. While there is nicotine-induced DA overflow among adolescent α6^CC^ and α6^GG^ males, there is no genotype difference in animals pretreated with saline or nicotine. However, we observe a genotype difference for METH-induced DA overflow in adolescent male saline-pretreated α6^CC^ and α6^GG^ rats. In contrast, we observe a more pronounced genotype difference in saline pretreated α6^CC^ females for both nicotine- and METH-induced DA release when compared to α6^GG^ females. Notably, α6^CC^ females exhibit greater DA overflow in response to nicotine and METH compared to α6^GG^ females particularly in the saline pretreatment condition. These results are in accordance with a previous study reporting acute nicotine increases DA transmission while long-term nicotine exposure reduces the release of DA in the NAc (Perez et al., 2012). In addition, the significant main and interactive effects of drugs indicate that nicotine and METH differentially influence DA overflow and its metabolites depending on genotype, sex, and pretreatment.

There is a significant difference in DOPAC levels based on genotype and sex, with α6^CC^ females showing higher levels of DOPAC in response to saline and nicotine pretreatments compared to α6^GG^ females, suggesting that genotype influences DA metabolism differently in females. In males, this genotype effect is not evident under acute conditions but becomes apparent under sub-chronic conditions with METH treatment, where α6^GG^ males exhibit higher DOPAC levels than α6^CC^ males. In the acute condition, α6^CC^ females exhibit higher DOPAC overflow for saline and nicotine compared to α6^GG^ females (*p* < 0.001), a difference not observed in males, while in the sub-chronic condition, α6^GG^ males show greater METH-induced DOPAC overflow compared to α6^CC^ males (*p* < 0.001). DOPAC levels suggest that the DAT, which is responsible for reuptake of DA from the synapse, may be influenced by genotype, sex, and drug exposure. Higher DOPAC levels in response to saline and nicotine pretreatments in α6^CC^ females compared to α6^GG^ females could indicate differences in DA reuptake or metabolism related to the DAT. Similarly, the differences observed in males under sub-chronic conditions with METH treatment suggest a potential role of the DA transporter in mediating these effects. Furthermore, the analysis of HVA levels revealed significant main and interactive effects influenced by genotype, sex, and pretreatment, as well as their interactions with drugs. Specifically, HVA overflow was altered in saline- and nicotine-treated α6^CC^ females compared to α6^GG^ females, an effect not observed in males. These results suggest that DA metabolism to DOPAC and HVA in the brain varies depending on the genetic background, sex, and prior exposure to substances like nicotine in female rats. The variations in HVA levels further suggest that the DA transporter may play a role in modulating DA metabolism in response to genetic and environmental factors. The altered HVA overflow in saline- and nicotine-pretreated α6^CC^ females compared to α6^GG^ females, specifically, could indicate differences in DA reuptake or metabolism that are influenced by the DAT. This variability in DA metabolism could potentially have implications for understanding how these factors contribute to differences in behavior or susceptibility to certain conditions. Future research that centers on the DA transporter has the potential to reveal additional insights into its function in these processes and its possible impact on behavior and vulnerability to specific conditions. Such studies could expand on these discoveries to develop a more thorough comprehension of how genetic and environmental factors influence DA metabolism and how these influences contribute to behavior and susceptibility to certain conditions.

Some potential limitations of our studies include the lack of assessment of drug-induced neurotransmitter release during METH self-administration. Given the technical difficulty of coupling *in vivo* microdialysis with METH self-administration, we have instead used a method that provides a potential readout of what our self-administration studies could provide if neurotransmitter release was simultaneously assessed during behavior. Given that contingent as compared with non-contingent delivery of drugs could differentially impact drug-induced neurotransmitter release, future studies could aim to couple these techniques with either *in vivo* microdialysis and/or fiber photometry to better understand the temporal dynamics of drug-induced neurotransmitter release and behavior. Further, our self-administration studies focus primarily on reinforcement behavior to METH at a fixed ratio one schedule of reinforcement. Thus, future studies could look at different schedules of reinforcement (e.g., fixed ratio 2-5), extended access and/or progressive ratio schedules of reinforcement, as well as extinction or reinstatement schedules of reinforcement, in order to determine how our findings impact other parameters of METH self-administration. Finally, while our studies focus on drug associated rewards, we could also evaluate whether our results impact natural rewards. While this is unlikely, given prior results related to this in wild type rats (Mojica et al., 2014) and the *CHRNA*6 SNP rats (Carreño and Lotfipour, 2023; Carreño et al., 2024), it would be an important question to assess in *CHRNA*6 3′UTR SNP rats in order to confirm that drug, not natural, rewards are what are most impacted by adolescent nicotine exposure.

## 5 Conclusion

The study investigated the effects of adolescent nicotine exposure on METH self-administration and DA overflow in the NAc shell of *CHRNA*6 3′-UTR SNP knock-in rats. The findings revealed significant interactions between sex, genotype, and nicotine pretreatment in modulating drug-induced DA overflow, metabolites, and METH self-administration in these rats. Nicotine pretreatment enhanced discrimination for reinforced non-reinforced responding for METH self-administration in α6^CC^ females rats, whereas saline-treated, but not nicotine-treated α6^GG^ females showed this type of discrimination for METH self-administration. Additionally, in male adolescents, prior nicotine exposure increased subsequent METH self-administration, maintenance, and intake, independent of the *CHRNA*6 3′-UTR SNP genotype. Female α6^CC^ rats showed higher DA overflow in response to nicotine and METH compared to α6^GG^ females. Male α6^GG^ rats exhibited higher METH-induced DA overflow than α6^CC^ males under sub-chronic pretreatment conditions, α6^CC^ females had higher DOPAC levels after saline and nicotine pretreatments than α6^GG^ females, indicating genotype influences DA metabolism differently in females. Significant differences in HVA levels were found based on genotype, sex, and pretreatment, with altered HVA overflow in saline- and nicotine pretreated α6^CC^ females compared to α6^GG^ females. In males, this effect appeared under sub-chronic METH conditions. The study suggests that the dopamine transporter plays a crucial role in modulating DA metabolism, with implications for behavior and susceptibility to conditions based on genetic and environmental factors. Understanding the complex interactions between sex, genotype, and nicotine exposure in modulating drug-induced behaviors and DA overflow can contribute to the development of more effective prevention and intervention strategies in the future. This can pave the way for more customized combined treatments such as pharmacotherapy, psychotherapy, and education to counter or lessen addiction behavior. Unlike conventional addiction methods that often follow a one-size-fits-all model with little consideration of a person’s genetics, personalized approaches can significantly improve treatment outcomes by tailoring interventions to an individual’s genetic profile and specific needs.

## Data Availability

The original contributions presented in the study are included in the article/supplementary material, further inquiries can be directed to the corresponding author.
